# Ten years of dynamic consent in the CHRIS study: informed consent as a dynamic process

**DOI:** 10.1038/s41431-022-01160-4

**Published:** 2022-09-05

**Authors:** Deborah Mascalzoni, Roberto Melotti, Cristian Pattaro, Peter Paul Pramstaller, Martin Gögele, Alessandro De Grandi, Roberta Biasiotto

**Affiliations:** 1 Institute for Biomedicine, Eurac Research, Affiliated Institute of the University of Lübeck, Bolzano, Italy; 2 Center for Research Ethics and Bioethics, Department of Public Health and Caring Sciences, Uppsala University, Uppsala, Sweden; 3 Department of Biomedical, Metabolic and Neural Sciences, University of Modena and Reggio Emilia, Modena, Italy

**Keywords:** Ethics, Genetics research

## Abstract

The Cooperative Health Research in South Tyrol (CHRIS) is a longitudinal study in Northern Italy, using dynamic consent since its inception in 2011. The CHRIS study collects health data and biosamples for research, and foresees regular follow-ups over time. We describe the experience with the CHRIS study dynamic consent, providing an overview of its conceptualization and implementation, and of the participant-centered strategies used to assess and improve the process, directly linked to participation and communication. In order to comply with high ethical standards and to allow broadness in the areas of research, CHRIS dynamic consent was conceived as an interactive process: based on a strong governance and an ongoing tailored communication with participants, it aims to promote autonomy and to develop a trust-based engaged relationship with participants, also relevant for retention. Built within an online platform, the consent allows granular choices, which can be changed over time. In a process of co-production, participants views have been investigated and kept into account in policy development. Participants showed a high degree of participation, thus enabling the consolidation of the CHRIS resources. Even though a low change rate was reported in the baseline, participants valued the possibility of changing their informed consent choices. Communication (language-tailored, ongoing, multimedia) was important for participants, and for participation and retention. In our experience, dynamic consent was proven to be a flexible consent model, which allowed to meet ethical and legal standards for participation in research, and to accommodate participants’ and researchers’ needs.

## Introduction

Dynamic consent is an informed consent model which enables research participants to revise and change their choices on their participation in research over time. Dynamic consent consists of an interactive platform and an ongoing communication approach that keeps participants informed on the development of the study over time and allows control on their end. Dynamic consent is considered to positively affect both recruitment and retention of participants, and the effective management of the informed consent process, particularly in biobanking and longitudinal studies where future uses of data and samples may not have been established at the time of recruitment [1, 2]. Dynamic consent has been proposed as a solution to meet both the ethical and legal requirements for scientific research and the expectations of researchers in the use of the samples and data [1, 2]. In the present paper, we describe the experience of the Cooperative Health Research in South Tyrol (CHRIS) study, the first population-based biobanking cohort study to use dynamic consent since its inception in 2011 [3]. The CHRIS study uses a participant-centered approach to build a relationship between researchers and participants [4]. We describe the conceptualization and the design of the CHRIS dynamic consent, the informed consent, the embedded communication strategy, and the participant-centered strategies for an ongoing assessment and improvement of the dynamic consent. We reflect on the impact of dynamic consent on participation and communication, and offer some reflections on the theoretical criticisms of dynamic consent from our practical experience of implementation. The logic framework for evaluation of dynamic consent [5] inspired the way we presented our experience with the CHRIS dynamic consent: the focus of the paper on participation, communication and engagement, and on what was achieved in those areas after its use for 10 years, provided an internal assessment respect to the purpose and assumptions underlying the design of the CHRIS dynamic consent.

## CHRIS study and dynamic consent conceptualization

### The CHRIS study

CHRIS is a longitudinal study based in the Val Venosta/Vinschgau district, a rural area in South Tyrol (Italy) focusing on the interplay between genetics, the environment and personal lifestyles on the human susceptibility and resilience to chronic conditions, specifically of the neurological, cardiovascular, and metabolic systems and on age-related health in general. The study has been collating a wealth of data from both direct measurement as well as questionnaire-based phenotyping, genetic and molecular characterization, pedigree reconstruction and bio-banked blood and urine specimens for future analyses [3, 6–8]. The CHRIS study aims to generate knowledge for the prevention and treatment of diseases and also promote health literacy in the population. The CHRIS closed cohort consists of 13,393 participants, recruited during the period 2011–2018 (recruitment phase) The first follow-up phase started in 2019, but was suspended until 2021 due to the COVID-19 emergency. Biological samples are stored in the biobank structures located in Bolzano/Bozen and Merano/Meran for backup, while data are securely and safely stored in internal servers and are regularly being backed up.

### Intersection of requirements and needs

In order to understand the conceptualization of the CHRIS dynamic consent, we provide here an overview of the context where it found its genesis.

The Italian Privacy Code passed in 2003 [9] set strict rules (in line with the European General Data Protection Regulation 2016/679 [10]), that also affected consent in research: specific consent and renewed consent for new studies became a requirement. In 2002–2003, Eurac Research conducted a population study focused on microisolates in South Tyrol, MICROS, which “was intended as the first step of a comprehensive approach for the assessment of the genetics of diseases affecting the South Tyrolean population” [11]. MICROS was the forerunner of what became the CHRIS study: scientists wanted to design a longitudinal, long-lasting population study, that would allow to efficiently conduct genetic and genomic research. The then new legal requirement imposed to identify an ethically sound, legally compliant consent model that could meet the needs of researchers conducting genetic and genomic research.

An empirical work, and an in-parallel theoretical analysis, enabled the evaluation of stakeholders’ needs (researchers, participants, ethics board), while keeping into account the features of the study, the type of data collected, and the ethical and legal implications related to the nature of the study [12]. To achieve the aims of the CHRIS study, researchers designed a population study which consisted in an extensive collection of sensitive data (genetic data, genealogical information, health-related and life-style data including alcohol and drugs consumption and psychiatric information) and follow-up plans in a long-term project. Follow-ups were deemed to be hampered by demanding and pricy logistics, and re-consent was regarded as too costly and burdensome. Other biobank studies in Europe opted for broad consent, but for the specific context of the CHRIS study, this was found unsuitable from both the Italian legal situation and from the ethical perspective. Given the extensive and sensitive nature of the data collected, in order to responsibly conduct a prospective study in the respect of participants, transparent and as complete as possible information about possible future uses of data and samples should have been provided. On a very utilitaristic note, ongoing contact over time and a trust-based relationship with research participants were deemed to be key elements for establishing a long-lasting relationship with the community which would positively affect participation and retention.

In this context, the solution to meet both the ethical and legal requirements (transparency, specific information, specific consent and re-consent) and the needs of researchers (long-term study, extensive collection of data and samples, broad aim of research and use of data and samples) was found with the development of a consent model which was defined as “interactive” [4], consisting of an ongoing communication process, thus allowing an “open dialogue” [13, 14] between participants and researchers, and “[*to incorporate*, Ed] community feedback into the policy frame of continued research” [13]. The dynamic consent of the CHRIS study was then structured in two main components: the consent form and the ongoing information supporting the consent [3]. With this participant-centered approach, the CHRIS dynamic consent was designed to develop a partnership with the study participants [4]. Therefore, the concepts that are found at the core of the CHRIS study’s dynamic consent come from a reflection conducted and developed since the early 2000s [12–14], suggesting that the informed consent model of the CHRIS study was conceptualized as dynamic even before the concept of dynamic consent became widely discussed in the literature [15].

## The CHRIS dynamic consent model

### The informed consent

The conceptual basis of the CHRIS informed consent is broad as regards the study aims, that is consent within predefined research areas (cardiovascular, neurological, metabolic, and oncologic health), and very specific with regard to the policy of the project for using data and samples (such as access rules, sharing rules, information policy, oversight) and participant rights [3, 14]. The broadness of the consent is then “informed” through an ongoing communication strategy.

The consent consists of an online onscreen interface, which is administered for the first time to each participant at the study center at the time of the baseline visit. In order to participate, participants must express their choice for all the questions of the informed consent. For some questions, which are not changeable over time, answering “Yes” is necessary for participation (Table 1). If a participant chooses “No”, the study assistant provides clarifications and explains why consent to the specific question is necessary for participating in the study (i.e., “Q1. I will participate in the following research program: blood sampling (for clinical laboratory analysis and DNA extraction); urine collection; weight and height measurement; blood pressure measurement; […]”; “Q6. I am aware that family trees will be created […]”). If the participant chooses “No” even after the explanation, they will not be able to participate. These questions also serve as further confirmation that participants understood concepts at the base of participation and to provide explanations where those concepts are not fully comprehended. The online consent provides several options, which can be monitored and changed over time. This can be done by accessing the consent through online authentication in the password-protected area MyCHRIS at any time, or by telephone contact with the CHRIS study center [3, 16]. This feature allows participants to visualize, confirm or review their preferences about involvement in research, acknowledging their right to change opinion. Some options of the consent are layered and structured with multiple choices. Participants having the possibility of choosing among different degree of participation was considered to better meet participants’ values and wishes, e.g., options for the return of results, and the data handling in case of death or incapacity (Table 1). Complete withdrawal from the study is always possible by contacting the study center.

**Table 1:** Choices in the informed consent of CHRIS baseline.

	Options	Type of choice
Q1. Consent to the visit	Yes	“Yes” is necessary for participation
	No	
Q2. Consent to data and samples processing	Yes	“Yes” is necessary for participation. Changeable upon withdrawal
	No	
Q3. Consent to data storage	Yes	“Yes” is necessary for participation. Changeable upon withdrawal
	No	
Q4a. Consent to data sharing with defined partners	Yes	Free-choice answer and changeable over time
	No	
Q4b. Consent to data sharing through portals	Yes	Free-choice answer and changeable over time
	No	
Q5. Options for medical reports delivery	At the CHRIS study	One option is necessary for participation
	Sent at home	
Q6. Awareness of pedigree study	Yes	“Yes” is necessary for participation
	No	
Q7. Consent to re-contact	Yes	Free-choice answer and changeable over time
	No	
Q8. Awareness of no economic benefit	Yes	“Yes” is necessary for participation
	No	
Q9. Consent to biobanking	Yes	“Yes” is necessary for participation. Changeable upon withdrawal
	No	
Q10. Death dispositions options	Destruction of data and samples	Free-choice answer and changeable over time
	Anonymisation of data and samples	
	Further use in research within the limit of the consent	
Q11. Awareness of research purpose	Yes	“Yes” is necessary for participation
	No	
Q12. Return of individual research results	Want to be informed	Free-choice answer and changeable over time
	Do not want to be informed	
	Informed only if results are relevant for own health and actionable	
	Informed only if results are potentially relevant for relatives’ health	

There are different types of questions in the informed consent: (a) questions where “Yes” is compulsory for participation and that are not changeable by the individual participant over time (Q1, Q2, Q3, Q5, Q6, Q8, Q9, Q11). However, some of those are changeable upon withdrawal (Q2, Q3, Q9); (b) questions where participants can choose among more options and that are changeable over time (Q4a, Q4b, Q7, Q10, Q12).

The informed consent conceived as dynamic promotes autonomy by providing participants with the possibility to easily change their perspectives over time, but also is a process that enables participants to express their preferences in accordance with changes to the research. For examples, with the first follow-up stage, changes were introduced in data collection, the process of consent to sub-studies and to be recruited for new studies, and the return of results policy [16, 17]. As an example, during the COVID-related suspension time of the follow-up phase, the CHRIS team started the CHRIS COVID-19 study, that aimed to understand the epidemiology and the effect of the SARS-CoV-2 infection on health [18]. Prospective participants were re-contacted through email or mail. For the first time, participation was extended to minors and cohabitants of CHRIS participants, and an entirely online informed consent (for the online survey study) was used. An information campaign through different channels supported the launch of the study. The online platform for consent, already established with the CHRIS dynamic consent, enabled the quick and smooth development and implementation of the CHRIS COVID-19 study, by contacting more than four thousand people in a brief period, responding timely to the health emergency demands. The data collected in the CHRIS COVID-19 study contributed to a large international genome-wide study aiming to understand the genetic factors associated with severity of the disease [19].

### Tailored ongoing communication

Dynamic consent can only work if based on dynamic communication. In CHRIS, the ethical, legal, social implications (ELSI)-based communication, i.e., the communication that forms the ethical basis of a choice, is regarded as essential. The culturally sensitive communication strategy was designed and has been implemented with a multilevel and multimedia approach. The development of this communication strategy took into account the intended targets, both the community and the individual, and was designed to allow a flow of information before, during and after the decision of participating in the CHRIS study [3] (Table 2).

**Table 2:** Multilevel and multimedia tailored communication.

Target	Media strategy	Intended aim of communication
Community	Meeting with stakeholders Public meetings Press releases	To engage the community To raise public awareness about the study
Individual	Before consenting Invitation letter Brochure One-page summary	To comply with transparency and to grant participant autonomy To inform on the study To raise awareness on the implication of participation
	At the CHRIS study center Movie explaining and summarizing the study Talk with the study assistant	
	After consenting Webpage MyCHRIS Newsletters Press releases Public conferences.	To dynamically inform on the development of the study To show the impact of the study To inform on the research that has been conducted To allow interaction between researchers and participants

In Val Venosta/Vinschgau, where the CHRIS study is conducted, German is the mother tongue for 97.3% of the population, while 2.6% of the population uses Italian as the main language [20]. Therefore, all the documents for the informed consent, the public engagement tools and the information materials have been produced in both German and Italian to guarantee the accessibility of the communication materials in the participant’s language of preference. As in Val Venosta/Vinschgau, the spoken German is a local dialect slightly different from the standard German, the team assistants, the medical doctors and the staff involved in collecting the data from the participants are fluent in the local dialect in order to allow an optimal understanding of the implications of participation and a comfortable experience for the participant. Participants can also express their preferences on means of re-contact (mobile phone, and/or home phone, and/or email) in their language of choice (i.e., German or Italian). As way of re-contact, large part of the CHRIS cohort provided the email address and mobile phone contact. Using a digital way of communication allows to decrease costs for re-contact for follow-ups and sub-studies.

### Participant engagement and dynamic consent

In line with the interactive communication approach, participant-centered strategies were used to assess and improve the dynamic consent with the inputs from participants. Participant views were elicited using qualitative and quantitative methods as well as field observations from actors in close contact with participants (study assistants, technical coordinators, researchers involved in the data collection). With these insights, participants views were incorporated into policy development as well as adjustments in the informed consent and the accompanying information material.

In order to assess the attitude towards online interface, before the beginning of the CHRIS study, a feasibility study aiming to compare the usability of computer-based and paper-based platform for survey questionnaire showed that participants preferred the computer-assisted modality over the paper-based one, and that they felt less nervous when using a computer, in comparison to a paper platform [3]. These findings assured the CHRIS study about the suitability of a dynamic IT-based interface.

The study assistants have been providing insightful observations about the informed consent process since the commencement of the study. The initial oral explanation of the study by the study assistants was replaced by a movie (around six months after the beginning of the study). The study assistants perceived an improvement in understanding about the study and the implications of participation, as since the introduction of the movie, there have been less questions and more situated requests of clarification. The introduction of the movie also had a beneficial impact on the overall enrollment rate as it has reduced the time required by the study assistants to explain the study to the participants from approximately 20 min to approximately 5 min [3]. This allowed to decrease the time and the costs associated with recruitment.

In the early stage of the recruitment phase (2012–2014), views on the dynamic consent were explored through semi-structured interviews and a survey with a subsample of CHRIS participants (Supplementary Information). This provided insights into participant satisfaction with the information provided and with the communication strategy, participant perception of the dynamic consent and satisfaction with some of its specific features, such as granularity of options, regular communication, and consent flexibility.

In 2018, the beginning of recall-by-genotype studies within the CHRIS study necessitated a change in the return of results policy. CHRIS participants were involved in this revision and empirical studies explored their views on the return of individual research results [17] and on the recall-by-genotype research approach [21]. The findings showed that participants want to make autonomous choices on the return of individual research result, and that a series of criteria affects their choice [17]. In light of these results, the informed consent question on the return of results changed by offering participants to express their choice on four types of results. These types of results are described through the example of four genetic diseases, chosen as representative of categories of risk and of treatment and prevention possibilities. This change in the informed consent and in the information provided to participants was applied in the follow-up phase. This process informed the refinement of the return of results policy and enabled a process of co-production of policy with the CHRIS participants.

## Observations coming from 10 years of experience

### Research participation

The CHRIS study is longitudinal in design, therefore the main goal of the CHRIS study in the recruitment phase was to maximize participation. The starting aim was to recruit at least 10,000 participants [3], but the population’s response was higher than expected and this figure was surpassed with ultimately 13,393 adult residents in Val Venosta/Vinschgau participating. The participation rate in two nested sub-studies conducted during the recruitment phase was also 77% and 86% [22, 23]. All the participants agreed to the data and samples storage in the biobank for 30 years for the research purposes of the CHRIS study (destruction of samples may be asked upon withdrawal). This provides the possibility of conducting long-term projects, of using the most innovative and suitable technologies available (at present or in the future) to process samples and conduct research and access to biological samples for the duration of the study.

In the informed consent, participants are given the possibility to choose different degrees of participation, as regards data sharing and leaving data and samples available for research in case of death or incapacity. Participants thus choose a participation level that best suits them over time within an established governance and oversight framework. The decisions that participants make regarding the extent of their participation are fundamental for the sustainability and success of a research study: in the CHRIS study, they may affect the completeness of the biobank data resource, the possibility of conducting research with international collaborators, and the whole long-term endeavor of a biobanking project. From the decade-long dynamic consent experience of the CHRIS study, we found that enabling participants to control the extent of their participation in research in the forms allowed by the dynamic consent proved to be beneficial for the study, because the majority of participants decided to agree to data sharing and to leave data and samples in case of incapacity or death (Table 3). Furthermore, in a survey with CHRIS participants conducted in 2014, participants highly endorsed the study and valued the possibility to change their consent choices over time, the possibility to choose among different options, and the ongoing regular communication (Supplementary Information, Table S2). This echoes the findings of other empirical studies that found that biobank participants’ preferences over samples availability changed over time, suggesting that dynamic consent, designed as an informed consent model that provides the strategy to address the possible changes in values and wishes that participants may experience through time, is relevant for participants [24]. Additionally, another empirical study with biobank participants investigating the experience of participating in biobanking research and of using a dynamic consent interface sample showed that the possibility of revising the consent given and the possibility of being informed about the research development through a dynamic consent tool represented for participants an opportunity for reciprocity and engagement [25].

**Table 3:** Choices in the informed consent. Absolute and relative frequency distribution of answers to selected items of the informed consent among the baseline CHRIS participants.

Decision	Option in the consent form	Yes *N* (%)	No *N* (%)
Data sharing	Sharing codified data with research partners (with binding data transfer agreement)	13,347 (99.69%)	42 (0.31%)
	Sharing codified data with institutions that allow data access to the scientific research community (through databases)	13,199 (98.58%)	190 (1.42%)
Handling of data and samples in case of death or incapacity	In case of death or incapacity, samples and data will be available for research within the limit described by the consent	11,574 (86.45%)	N/A
	In case of death or incapacity, samples and data will be anonymized	1161 (8.67%)	N/A
	In case of death or incapacity, samples and data will be destroyed	653 (4.88%)	N/A
Re-contact for information and research	Re-contact for receiving information or re-consent to sub-studies/new studies	13,343 (99.66%)	46 (0.34%)

Data about the dynamic consent choices which are relevant for participation (data sharing, handling of data and samples in case of death or incapacity) and communication (re-contact for information and research) are shown. In this table, only data of participants who agreed to data storage for research purposes and biobank conservation are considered (*N* = 13,389).

In the CHRIS baseline, 22 participants changed their choices in the informed consent in the period 2011–2018. The most common change was for the option related to re-contact with relevant secondary findings (14 changes), followed by the option on death or incapacity disposition (8 changes), the option on data sharing (partners 5, portals 4), the option on re-contact (2 changes). The changes were made at a minimum of 1 day and a maximum of 2069 days after providing their initial consent, with a median of 18 days. We expect that this scenario may change over time, as a reflection of the development and growth of the CHRIS study (e.g., possible increase of number of sub-studies, possible increase of data and samples sharing). The possibility of changing options will allow participants to change their decisions while the study itself changes. For example, in the baseline, almost all CHRIS participants agreed to data and samples sharing (Table 3), but it cannot be plainly assumed that their decision will stay the same over the years, while the CHRIS study develops and grows, and while data and samples sharing processes may increase thanks to the consolidation of the biobank resources and databases. Providing the possibility of choosing about data and samples sharing would be positive not only for the respect of participants but also for retention. In fact, there may be participants who no longer want to share their data and samples, but who are still interested in participating in the CHRIS study. In this way, they can continue to participate, and their data and samples can be used for projects conducted by internal groups, or used as aggregated data, resulting in a general benefit for research. It is possible that without this option, they would not have participated at all, or would have drop out at some point because participation no longer corresponded with their wishes.

### Communication with participants

Previous empirical studies showed that biobanking participants valued communication, information, and engagement [25, 26], and that the regular provision of information was important as a trust-building element [27, 28]. Additionally, multimedia approaches have been demonstrated to be beneficial for enhancing participant understanding [29]. Indeed, what characterizes the dynamic consent of the CHRIS study is the important role that communication with participants plays in the informed consent process. In a multilevel fashion, the multimedia communication developed through time—before, during and after the consent in participation—and aimed to engage both the community and the prospective participant. The majority of participants agreed to being re-contacted for receiving information and for participating in sub-studies (Table 3). A subsample of CHRIS participants also valued regular communication with the study and were satisfied with the information provided and with the multimedia approach in promoting the understanding of the study (Supplementary Information, Table S2). In our experience, a culturally sensitive communication characterized by the possibility of choosing the preferred language of communication, the availability of information in both languages, the interaction with professionals fluent in German, Italian and local German dialect, combined with a local initiative (collaboration among Eurac Research, the South Tyrol health system, the local general practitioners (GPs)) has been key for the development of a trust-based relationship with participants that results in a successful recruitment and retention.

Dynamic consent was criticized for its reliance on information technology, and the possible overlooking of the impact of the digital divide [30]. Inequality in the access to digital technology and exclusion have been raised as potential problems associated with a dynamic consent model [2, 30]. In the CHRIS study, the attention to communication included a reflection on access to digital technology and on digital literacy within the CHRIS cohort and the general population. Although the use of information technology in South Tyrol is progressively increasing and at a fast pace (for example, in 2018 79.2% of the South Tyrolean population used internet [31], compared to 59.9% in 2010 [32]), in 2018, when the cohort’s recruitment ended, it was still differentially distributed by age, with the eldest being the category least familiar with information technology [31]. Indeed, with the survey conducted in 2014 on dynamic consent, we found that older people were those that had the lower easiness with the digital platform. In the CHRIS study, we addressed these issues by using a multimedia communication approach, that also included conventional paper-based materials, and by offering the possibility of contacting by phone the CHRIS center for request of changes in the options in the consent form. In this way, even though the dynamic consent was developed as an online onscreen tool, we guaranteed a variety of approaches that best suits participants digital literacy. Additionally, when participants came at the CHRIS center for their participation, they had the possibility of asking the study assistants technical help for filling in the online consent form, thus transforming the visit at the CHRIS center as an opportunity for learning how to access the personal page and how to navigate the dynamic consent interface.

### Misconceptions about dynamic consent

In the theoretical debate on informed consent, it has been argued that participants’ ability to decide about the extent of their participation and about the use of their data and samples through dynamic consent may have a negative impact. There is the concern that dynamic consent will affect the research’s public good, impair governance, and that dynamic consent may affect the authority of governance and oversight bodies [33, 34]. Additionally, repeated re-consent requests and consent fatigue have been considered as drawbacks of dynamic consent [2, 30, 35]. In our view, based upon our decade-long experience of dynamic consent, we believe that these concerns mostly reflect a misconception of the practical application of dynamic consent, in particular, a misunderstanding of the role of governance and on the role of communication within dynamic consent. We previously addressed some of the criticism on dynamic consent: by clarifying the mechanism of data sharing and the management of consent and re-consent in the CHRIS study [16], we showed how participants exert their will dynamically within the clear boundaries defined by the strong governance in place in the CHRIS study. The dynamic concept at the base of CHRIS dynamic consent translates into a flexible adjustment to participants needs, aiming to promote autonomy, participant engagement and inclusivity within a solid ethical and legal framework set through defined governance structures. This occurs through ongoing communication designed to keep participants aware of the further development of the study. It does not overwhelm participants but rather respects their preferences for method and frequency of communication. In our example, the theoretical challenges do not materialize in practice when dynamic consent is ethically applied.

## Conclusion

In the present paper, we provided a description of the dynamic consent of the CHRIS study. We described its conceptualization, the communication strategy that is integral part of the dynamic consent, and participant involvement in co-production of policy and forms of research participation. With a decade-long experience with CHRIS dynamic consent, we aimed to shed light into the practical implementation of a discussed consent model, and to show the actual potentialities of dynamic consent as an ethical-legal tool for research participation in long-term research endeavors such as longitudinal studies. Considering that the CHRIS study has been using dynamic consent since its inception, we did not provide a formal assessment of outcomes in line with an evaluation framework. However, by sharing our experience with CHRIS dynamic consent, we hope to throw light into the nuances that the implementation of a dynamic consent model allowed to address and resulted in.

## Supplementary information

Understanding participant response to dynamic consent

## Data Availability

The datasets generated and analyzed during the current study are available in the CHRIS study repository. For application for CHRIS data, contact the CHRIS study access committee (access.request.biomedicine@eurac.edu). Further information on the CHRIS study is available at https://www.eurac.edu/en/institutes-centers/institute-for-biomedicine and https://it.chris.eurac.edu/.
