# Supplementary material for: Ten years of dynamic consent in the CHRIS study: informed consent as a dynamic process
Source: Eur J Hum Genet. 2022 Sep 5;30(12):1391–7. doi: 10.1038/s41431-022-01160-4 (PMC9441838; doi:10.1038/s41431-022-01160-4)
Supplement: Supplementary file 1 — Understanding participant response to dynamic consent [file 41431_2022_1160_MOESM1_ESM.docx]

# Supplementary Information:

# Understanding participant response to dynamic consent

## Methods

In order to understand participant perception of the informed consent process, we first adopted an exploratory qualitative approach, which allowed to inform the issues that were further analyzed through a quantitative approach. In the exploratory phase, 16 semi-structured interviews were conducted between December 2012 and March 2013 at the CHRIS center with CHRIS participants at the end of the data collection visit. We aimed to explore participants’ understanding of the informed consent and attitudes and views towards the features of dynamic consent. In detail, the dimensions which were explored are as follow: 1) the views on quality and quantity of information provided in the informed consent process; 2) the views on the features of the dynamic consent such as multiple-choice questions, and possibility of changing answers, and on the online personal space; 3) understanding of the informed consent questions; 4) attitude towards receiving information. Respondents’ age ranged between 21 and 73 years old, eight were female and eight were male.

### Design of the quantitative study

Between July 2014 and October 2014, we administered a paper-based quantitative survey to an opportunistic sample of the CHRIS participants, recruited prospectively among participants to the study visit. A total of 508 participants out of 511 (99.4%) invited to take part at the time of clinical examination filled in the questionnaire. The survey aimed to investigate participant views on CHRIS dynamic consent by addressing the following points: satisfaction with information; the role of multimedia communication in participant understanding; perceived usability (technological obstacle); role of consent granularity in perception of respect for values and will; value of regular communication and of consent flexibility and associated trust; level of endorsement of the study. The questionnaire included seven questions (Box S1). The first six questions (Q1-6) were completed immediately after the informed consent procedure, upon consenting to participation. Q7 was asked instead at the end of participation. For each question, participants replied with a score on a discrete numeric rating scale between 0 (completely disagree) and 10 (completely agree). All questions but Q3 were phrased positively.

**Box S1: Survey questions**.

| **Questions** |
| --- |
| Q1. I received all the information I sought.  Q2. Having received information by different means (letter, video, brochure, consultation with the nurse) helped me best to understand the study.  Q3. Filling-in the electronic informed consent on the computer was difficult.  Q4. Being able to choose between several options ensures that my will is respected.  Q5. The awareness that I am receiving regular information about the study gives me peace of mind.  Q6. Being able to change the consent granted gives me peace of mind.  Q7. I would recommend participation into the CHRIS study to a family member or friend. |

### Data Analysis

For each question of the survey, best rating was defined as scores of 9 or 10, except for Q3, which was asked as a negative statement, and therefore scores (0-1) represent best rating. Question responses were then compared according to sex, age group and education. Data are presented as counts within categories and percentages across categories. Chi square tests with 1 degree of freedom were used to obtain statistical evidence of any difference at the statistical nominal p-value of 0.05.

## Results

The socio-demographic characteristics of the survey respondents are shown in Table S1.

**Table S1: Socio-demographic characteristics of the 508 survey participants.**

| **Characteristics** | **N** | **%** |
| --- | --- | --- |
| **Sex** |  |  |
| Male | 222 | 43.7% |
| Female | 286 | 56.3% |
|  |  |  |
| **Age** |  |  |
| <40 | 207 | 40.7% |
| 40-59 | 199 | 39.2% |
| 60+ | 102 | 20.1% |
|  |  |  |
| **Education** |  |  |
| compulsory school | 154 | 30.3% |
| higher education | 354 | 69.7% |

Table S2 shows the results of the survey. As a whole, most respondents (more than 90%) highly endorsed the study (Q7) and were satisfied with the information received (Q1). The majority of participants (between 80 and 89%) reported that the multimedia approach improved their understanding (Q2), that consent granularity ensured respect of their will (Q4), and that consent flexibility was valuable for their peace of mind (Q6). Usability of the electronic informed consent (Q3) and the value of regular communication (Q5) were highly scored by a great part of respondents (between 70 and 79%).

We found some differences when answers were analyzed by sex, age group, and education. Female respondents reported the highest score in greater proportion than males when rating the role of multimedia approach in understanding, the value of consent flexibility, and the endorsement of the study (Q2, Q6, Q7). Younger respondents (below 40 years old) and with higher education were more satisfied with the received information and with the usability of the online interface than their respective counterparts (Q1, Q3). Older respondents (over 60 years old) and respondents with lower education valued regular communication more than their counterparts (Q5). Older respondents valued consent flexibility more than the younger ones (Q6).

### Limitations

Our survey used 6 positively phrased questions and 1 negatively phrased question. We acknowledge that this may have induced more positive than negative ratings by participants. In order to limit asymmetry of responses, we used a numeric rating scale of 11 points for the sake of accuracy, which was equally balanced at the anchored extremes. We also classified ‘best ratings’ as those scoring 9 or 10 for positive questions and 0 or 1 for the negative question, which made the basis of our comparisons. Therefore, we believe that we have limited the risk of biased responses to a reasonable extent.

**Table S2. Best rating**^§^ **to each survey question, by socio-demographic characteristic of participants.**

|  | **Sex** | | | |  | **Age group** | | | | | |  | **Education** | | | |  |  | |
| --- | --- | --- | --- | --- | --- | --- | --- | --- | --- | --- | --- | --- | --- | --- | --- | --- | --- | --- | --- |
|  | **Male** | | **Female** | |  | **<40** | | **40-59** | | **60+** | |  | **Compulsory school** | | **Higher education** | |  | **Total** | |
| **Question** | **N** | **%** | **N** | **%** | **p-value** | **N** | **%** | **N** | **%** | **N** | **%** | **p-value** | **N** | **%** | **N** | **%** | **p-value** | **N** | **%** |
| **Q1** |  |  |  |  |  |  |  |  |  |  |  |  |  |  |  |  |  |  |  |
| (0-8) | 27 | 12.2% | 22 | 7.7% | 0.091 | 13 | 6.3% | 20 | 10.1% | 16 | 15.7% | 0.030 | 22 | 14.3% | 27 | 7.6% | 0.019 | 49 | 9.6% |
| (9-10) | 195 | 87.8% | 264 | 92.3% |  | 194 | 93.7% | 179 | 89.9% | 86 | 84.3% |  | 132 | 85.7% | 327 | 92.4% |  | 459 | 90.4% |
| **Q2** |  |  |  |  |  |  |  |  |  |  |  |  |  |  |  |  |  |  |  |
| (0-8) | 43 | 19.4% | 33 | 11.5% | 0.014 | 26 | 12.6% | 27 | 13.6% | 23 | 22.5% | 0.053 | 30 | 19.5% | 46 | 13.0% | 0.060 | 76 | 15.0% |
| (9-10) | 179 | 80.6% | 253 | 88.5% |  | 181 | 87.4% | 172 | 86.4% | 79 | 77.5% |  | 124 | 80.5% | 308 | 87.0% |  | 432 | 85.0% |
| **Q3** |  |  |  |  |  |  |  |  |  |  |  |  |  |  |  |  |  |  |  |
| (2-10) | 52 | 23.4% | 75 | 26.2% | 0.470 | 21 | 10.1% | 48 | 24.1% | 58 | 56.9% | <0.001 | 62 | 40.3% | 65 | 18.4% | <0.001 | 127 | 25.0% |
| (0-1) | 170 | 76.6% | 211 | 73.8% |  | 186 | 89.9% | 151 | 75.9% | 44 | 43.1% |  | 92 | 59.7% | 289 | 81.6% |  | 381 | 75.0% |
| **Q4** |  |  |  |  |  |  |  |  |  |  |  |  |  |  |  |  |  |  |  |
| (0-8) | 43 | 19.4% | 41 | 14.3% | 0.130 | 36 | 17.4% | 32 | 16.1% | 16 | 15.7% | 0.908 | 25 | 16.2% | 59 | 16.7% | 0.904 | 84 | 16.5% |
| (9-10) | 179 | 80.6% | 245 | 85.7% |  | 171 | 82.6% | 167 | 83.9% | 86 | 84.3% |  | 129 | 83.8% | 295 | 83.3% |  | 424 | 83.5% |
| **Q5** |  |  |  |  |  |  |  |  |  |  |  |  |  |  |  |  |  |  |  |
| (0-8) | 67 | 30.2% | 73 | 25.5% | 0.244 | 80 | 38.6% | 42 | 21.1% | 18 | 17.6% | <0.001 | 32 | 20.8% | 108 | 30.5% | 0.024 | 140 | 27.6% |
| (9-10) | 155 | 69.8% | 213 | 74.5% |  | 127 | 61.4% | 157 | 78.9% | 84 | 82.4% |  | 122 | 79.2% | 246 | 69.5% |  | 368 | 72.4% |
| **Q6** |  |  |  |  |  |  |  |  |  |  |  |  |  |  |  |  |  |  |  |
| (0-8) | 54 | 24.3% | 47 | 16.4% | 0.027 | 52 | 25.1% | 34 | 17.1% | 15 | 14.7% | 0.044 | 27 | 17.5% | 74 | 20.9% | 0.382 | 101 | 19.9% |
| (9-10) | 168 | 75.7% | 239 | 83.6% |  | 155 | 74.9% | 165 | 82.9% | 87 | 85.3% |  | 127 | 82.5% | 280 | 79.1% |  | 407 | 80.1% |
| **Q7** |  |  |  |  |  |  |  |  |  |  |  |  |  |  |  |  |  |  |  |
| (0-8) | 29 | 13.1% | 19 | 6.6% | 0.014 | 17 | 8.2% | 17 | 8.5% | 14 | 13.7% | 0.254 | 15 | 9.7% | 33 | 9.3% | 0.882 | 48 | 9.4% |
| (9-10) | 193 | 86.9% | 267 | 93.4% |  | 190 | 91.8% | 182 | 91.5% | 88 | 86.3% |  | 139 | 90.3% | 321 | 90.7% |  | 460 | 90.6% |
| **Total** | **222** | **100.0%** | **286** | **100.0%** |  | **207** | **100.0%** | **199** | **100.0%** | **102** | **100.0%** |  | **154** | **100.0%** | **354** | **100.0%** |  | **508** | **100.0%** |

Symbols and abbreviations: N represents cell counts; % are column percentages.

P-values were obtained by the chi-square test for each comparison among categories.

^§^For each question, the best rating was defined as scores of 9 or 10, except for Q3, which was asked as a negative statement, and therefore scores (0-1) represent best rating.
